# Extracting Concrete Thermal Characteristics from Temperature Time History of RC Column Exposed to Standard Fire

**DOI:** 10.1155/2014/242806

**Published:** 2014-08-11

**Authors:** Jung J. Kim, Kwang-Soo Youm, Mahmoud M. Reda Taha

**Affiliations:** ^1^School of Architecture, Kyungnam University, Changwon-si 631-701, Republic of Korea; ^2^GS E&C Research Institute, Gyeonggi-do 449-831, Republic of Korea; ^3^Department of Civil Engineering, University of New Mexico, Albuquerque, NM 87131-0001, USA

## Abstract

A numerical method to identify thermal conductivity from time history of one-dimensional temperature variations in thermal unsteady-state is proposed. The numerical method considers the change of specific heat and thermal conductivity with respect to temperature. Fire test of reinforced concrete (RC) columns was conducted using a standard fire to obtain time history of temperature variations in the column section. A thermal equilibrium model in unsteady-state condition was developed. The thermal conductivity of concrete was then determined by optimizing the numerical solution of the model to meet the observed time history of temperature variations. The determined thermal conductivity with respect to temperature was then verified against standard thermal conductivity measurements of concrete bricks. It is concluded that the proposed method can be used to conservatively estimate thermal conductivity of concrete for design purpose. Finally, the thermal radiation properties of concrete for the RC column were estimated from the thermal equilibrium at the surface of the column. The radiant heat transfer ratio of concrete representing absorptivity to emissivity ratio of concrete during fire was evaluated and is suggested as a concrete criterion that can be used in fire safety assessment.

## 1. Introduction

Fire safety for structural members in buildings is necessary to be assessed as failure of structural members due to fire can cause extensive property damage or loss of life. Many research efforts have been conducted to assess fire safety for structural members [1–3]. For a reinforced concrete (RC) member exposed to fire, the increase in temperature in both steel and concrete causes a decrease in strength and stiffness of the member. Therefore, most design codes for RC structures limit temperatures in RC member subject to fire [4–8]. Alternatively, effective sections can be considered for fire safety assessment of RC members such that the strength of RC member under fire is evaluated with a reduced strength for reinforcing steel based on its temperature while the strength of concrete over 500°C is ignored [7, 8]. Temperature variations in the RC member exposed to fire depends on the thermal properties of concrete and steel and the shape of the cross-sections. The thermal properties of concrete and steel are obtained from fire test or using prediction methods. While many methods were proposed to predict temperature variations in concrete members subject to fire [9–13], there exists difficulty to choose the thermal characteristics, especially thermal conductivity of concrete. This work suggests a simple method to identify thermal characteristics of concrete under fire.

The thermal conductivity of concrete is a major property that affects the temperature variations in concrete members subject to fire. In normal concrete, the thermal conductivity varies from 0.5 W/(mK) to 3.3 W/(mK) depending on the type of aggregate and the concrete mixture used [15–19]. As the thermal conductivity of concrete is relatively lower than that of other construction materials such as 53.3 W/(mK) at 20°C for structural steel [20], RC members exhibit good performance in fire if extensive spalling is prevented [14, 21]. The thermal conductivity of concrete has also been reported to be affected by the measuring techniques [19, 22–24]. To measure the thermal conductivity of concrete at a test temperature, it requires the concrete specimen to reach the status of thermal steady-state for the test temperature [23, 24]. As the thermal conductivity of concrete is relatively low, it takes significantly long time for a concrete brick to reach the status of thermal steady-state for a test temperature which is typically a high temperature. For example, the soak period of concrete bricks (230 mm × 114 mm × 65 mm) for a test temperature of 600°C is four days. This relatively long time-period is necessary to measure the thermal conductivity of concrete following ASTM C1113 [14]. If a test time period shorter than the ASTM standard is to be used, it would be necessary to develop a method to measure thermal conductivity from temperature variations in RC under unsteady-states condition.

The heat flux is transferred from one medium to the other by conduction, convection, and radiation [25]. In general, the heat flux is transferred from fires to structures by both convection and radiation [26, 27]. The convective heat flux is proportional to the difference in temperatures between the convection currents due to fire and the structural surface [26, 27]. The radiant heat flux of fire will be transferred to the surface of a structure by absorptivity ratio of the surface while the structure emits the radiant heat flux in proportion to its emissivity [28]. The transferred heat flux from a fire to the structure is then transferred into the structure by conduction. In fire tests of RC structures, the thermal steady-state condition when the temperatures of gas in fire test furnaces and the whole body of test structure become equal has been rarely observed [11]. However, if a decreasing temperature change with time is observed, at the surface of RC structure during a fire test using a standard fire, the thermal radiation properties of RC structure might be estimated by considering thermal equilibrium of the surface.

In this study, we suggest an inverse methodology to identify thermal conductivity of concrete from time history of temperature variations during fire test. The developed methodology was applied to find the thermal conductivity of RC column exposed to a standard fire. Fire test of an RC column was conducted following ISO-834 standard fire curve [29]. The profiles of time history of temperatures at six different locations across the RC column section were obtained. The thermal conductivity of RC columns was then determined by minimizing errors between the numerical solution and the temperature observations with time. The determined thermal conductivity was compared to that observed from experiments following ASTM C1113 [24] for verification of the proposed method. Moreover, the thermal radiation properties of the RC column were estimated considering the thermal equilibrium at the surface of the RC column in fire. The use of the thermal radiation properties of RC columns as a fire safety index is discussed.

## 2. Experimental Methods

**Figure 1:**
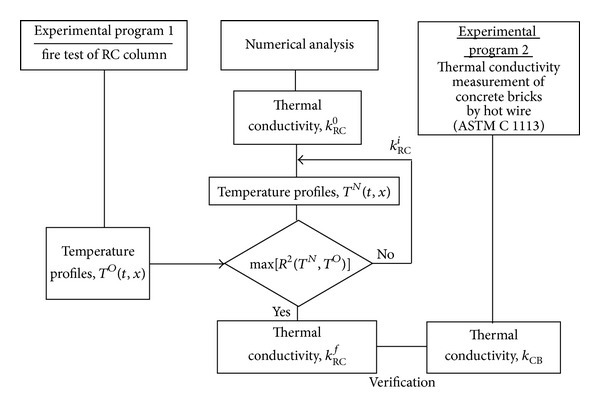
Layout of the proposed research program to extract thermal conductivity from unsteady-state test measurements and verify the measurements using standard test methods.

In this work, two experimental programs were conducted as shown in Figure 1. First, fire test of an RC column was prepared to obtain the time history of temperature variations in a section of the column. The temperature variations will be used to extract the thermal conductivity of the column using a proposed numerical method. To verify the results of the thermal conductivity extracted from RC column testing, the second experimental program to measure the thermal conductivity of concrete bricks was conducted following ASTM C1113 [24].

### 2.1. Fire Test of RC Column

**Figure 2:**
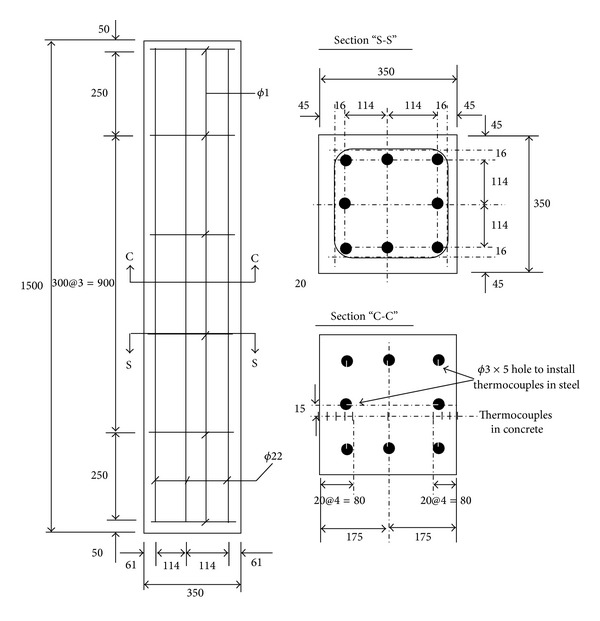
Details of RC column for fire test and locations of thermocouples. All dimensions are in mm.

**Figure 3:**
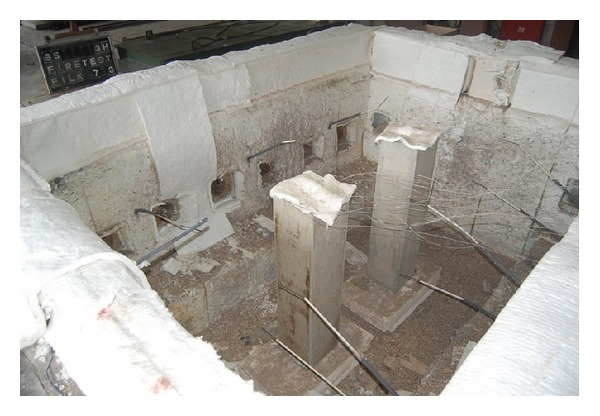
RC columns in furnace before fire test.

An RC column was tested to evaluate the time history of temperature variations following ISO-834 standard fire curve [29] for 180 minutes. The ISO-834 standard fire curve is presented as

(1) $$\begin{array}{c} T_{g}=345\log\left(8t+1\right)+T_{0}\;\;\left[{}^{{}^{\circ}}\text{C}\right], \end{array}$$

where *T*_*g*_ is the gas temperature in fire test furnace at time *t* in minutes. *T*_0_ is the ambient temperature. The RC column was constructed following the design specification proposed by the Korean Concrete Institute (KCI) [30]. The RC column had the square section of 350 mm by 350 mm and the height of 1500 mm. The column was reinforced with 8-*ϕ*22 mm steel rebars longitudinally and *ϕ*10 mm hoop bars placed at 300 mm spacing along the column height. Grade 420 steel rebars, which have the yield strength of 420 MPa, were used to reinforce the column. Details of column reinforcement are shown in Figure 2. A concrete having the 28-day characteristic compressive strength of 39.9 MPa was used for the column. The maximum aggregate size of 25 mm pea gravel was selected for the concrete. The slump of fresh concrete was controlled in the range from 180 mm to 220 mm. The mixture proportion of the concrete is presented in Table 1. The temperatures in concrete following the standard fire curve were measured at the center section of the column located at 750 mm from the bottom of the column. Across the section of the column, temperature was measured at the surface, 20 mm, 40 mm, 60 mm, 80 mm, and 175 mm from both sides, respectively. The temperatures in longitudinal steel rebars were also measured for all 8 rebars at the center section where the temperatures of concrete were measured. The locations of the thermocouples are shown in Figure 2. The column was placed in fire test furnace as shown in Figure 3 and the time histories of temperatures following the standard fire curve for 180 minutes were recorded.

### 2.2. Thermal Conductivity Measurement of Concrete Bricks

**Figure 4:**
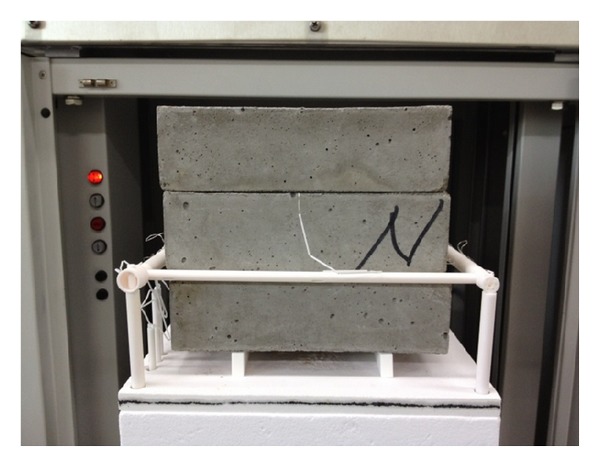
Thermal conductivity measurement of concrete bricks (230 mm × 114 mm × 65 mm) using hot wire (platinum resistance thermometer technique) [14].

To verify the proposed inverse method to identify thermal conductivity, a standard test method to measure thermal conductivity of concrete was used as shown in Figure 4. In this standard method, the thermal conductivity of concrete bricks constructed with the mixture proportion presented in Table 1 was measured following ASTM C1113 [24]. For the main part of the harness wire, platinum wire of 0.33 mm diameter was used. The specimens consisted of three 230 mm × 114 mm × 65 mm straight concrete bricks. The thermal conductivity of concrete bricks was measured at nine test temperatures of 50°C, 100°C, 200°C, 300°C, 400°C, 500°C, 600°C, 670°C, and 780°C. The thermal conductivity of concrete bricks was measured three times at each test temperature. Heating rate for the furnace temperature was 55°C/hour. Soak periods to make concrete bricks reach thermal steady-state with the test temperature were different for each test temperature.

## 3. Numerical Methods

In a thermal system without the heat generation, one-dimensional unsteady-state heat equation considering both specific heat and thermal conductivity as functions of temperature can be formulated as

(2) $$\begin{array}{c} \rho \frac{\partial \left\{c\left(T\right)T\left(t,x\right)\right\}}{\partial t}=\frac{\partial }{\partial x}\left\{k\left(u\right)\frac{\partial T\left(t,x\right)}{\partial x}\right\}, \end{array}$$

where *T*(*t*, *x*) is the temperature of concrete at the distance *x* from the surface at time *t* and *ρ* is the density of concrete considered constant and equal to 2300 kg/m^3^ in all calculations. *c*(*T*) and *k*(*T*) are the specific heat and the thermal conductivity of concrete at temperature *T*. Equation (2) can be expanded as

(3) ρ{c(T)+T(t,x)dc(T)dT}∂T(t,x)∂t =k(T)∂2T(t,x)∂x2+dk(T)dT(∂T(t,x)∂x)2.

For a finite difference approximation, all variables in (3) except the temperature *T* are linearly transformed to be dimensionless by putting $\hat{x}=x/l^{*}$, $\hat{t}=t/t^{*}$, $\hat{k}(T)=k(T)/k^{*}$, and $\hat{c}(T)=c(T)/c^{*}$. Equation (3) is then rearranged as

(4) ∂T(t^,x^)∂t^=k∗t∗ρc∗(l∗)2 ×[k^(T)(∂2T(t^,x^)/∂x^2)+(dk^(T)/dT)(∂T(t^,x^)/∂x^)2][c^(T)+T(t^,x^)(dc^(T)/dT)].

Putting t* = ρc*(l*)^2^/k*, the constant term in the right side of (4) becomes one. A finite difference approximation of (4) will be expressed as

(5) T(i+1)j=Aij{Ti(j+1)−2Tij+Ti(j−1)} +Bij{Ti(j+1)−Tij}2+Tij,

where

(6) $$\begin{array}{c} A_{ij}=\frac{\Delta t}{\Delta x^{2}}\frac{{\hat{k}}_{ij}}{{\hat{c}}_{ij}+{\hat{c}}_{ij}^{\prime }u_{ij}},\\ B_{ij}=\frac{\Delta t}{\Delta x^{2}}\frac{{\hat{k}}_{ij}^{\prime }}{{\hat{c}}_{ij}+{\hat{c}}_{ij}^{\prime }u_{ij}}, \end{array}$$

where *i* is the number of time steps and *j* is the number of distance steps. Δ*t* and Δ*x* are the step sizes for time and distance, respectively. ${\hat{k}}_{ij}$ and ${\hat{k}}_{ij}^{\prime }$ are the thermal conductivity and the corresponding derivative with respect to temperature evaluated at the temperature of *T*_*ij*_ while ${\hat{c}}_{ij}$ and ${\hat{c}}_{ij}^{\prime }$ are the specific heat and the corresponding derivative with respect to temperature evaluated at the temperature of *T*_*ij*_. Using the quadratic forms of the thermal conductivity and the specific heat [6] as

(7) k(T)=α{(T120)2−20(T120)+200}20°C≤T≤1200°C  [W/(mK)]

(8) c(T)=β{225+20(T120)−(T120)2}20°C≤T≤1200°C  [J/(kg°C)],

the coefficients *A*_*ij*_ and *B*_*ij*_ in (5) can be calculated as

(9) $$\begin{array}{c} A_{ij}=\frac{\alpha }{\beta }\frac{\Delta t}{\Delta x^{2}}\frac{c^{*}}{k^{*}}\frac{288\times 10^{4}-2400\;T_{ij}+T_{ij}^{2}}{324\times 10^{4}+4800\;T_{ij}-3T_{ij}^{2}},\\ B_{ij}=\frac{\alpha }{\beta }\frac{\Delta t}{\Delta x^{2}}\frac{c^{*}}{k^{*}}\frac{2T_{ij}-2400}{324\times 10^{4}+4800\;T_{ij}-3T_{ij}^{2}}. \end{array}$$

The normalizing factors k* and c* for the thermal conductivity and the specific heat are selected as those evaluated at 20°C using (7) and (8), respectively. While *β* for the specific heat in (8) is fixed at 4.0 [6], *α* for the thermal conductivity in (7) will be changed to find the calculated time history of temperature variations which gives the minimum error with the observed time history of temperature variations from the RC column exposed to the standard fire. The founded *α* will be used to determine the thermal conductivity of concrete as a quadratic function of temperature using (7).

## 4. Results and Discussion

### 4.1. Thermal Conductivity of Concrete

**Figure 5:**
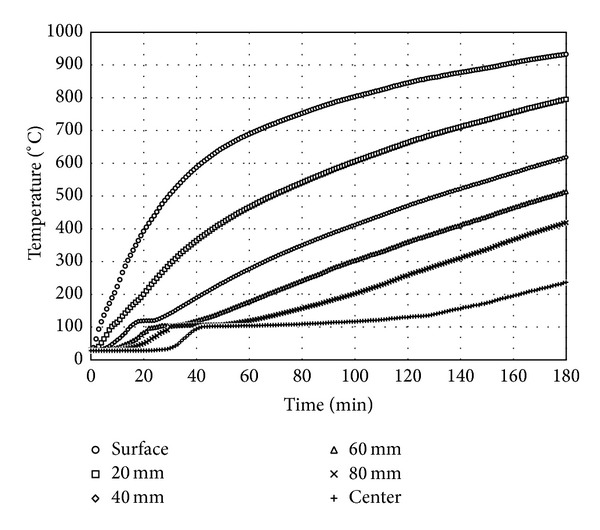
Temperature variations with respect to time in concrete.

After finishing the fire test of the RC column for 180 minutes, it was confirmed that there was no spalling of the RC column. The time histories of temperatures measured in concrete at the two locations for the same depth from the concrete surface are averaged and presented in Figure 5 for the depths of 0 mm (surface), 20 mm, 40 mm, 60 mm, 80 mm, and 175 mm, respectively. For the time histories of temperatures at 40 mm, 60 mm, 80 mm, and 175 mm in Figure 5, the temperature increases stop at the temperature of 100°C for a while and then continue. This can be attributed to the effect of capillary water on concrete thermal behavior. For normal concrete, it would have partially saturated capillary pores. This enables concrete to conduct heat rapidly up to the temperature of 100°C. For temperatures above 100°C, water in the capillary pores evaporates and the concrete with empty pores will conduct heat more slowly than that with partially saturated pores.

**Figure 6:**
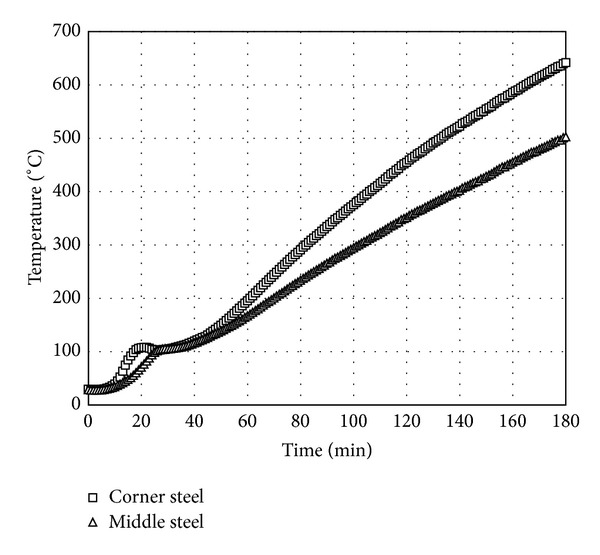
Temperature variations with respect to time in steel.

**Figure 7:**
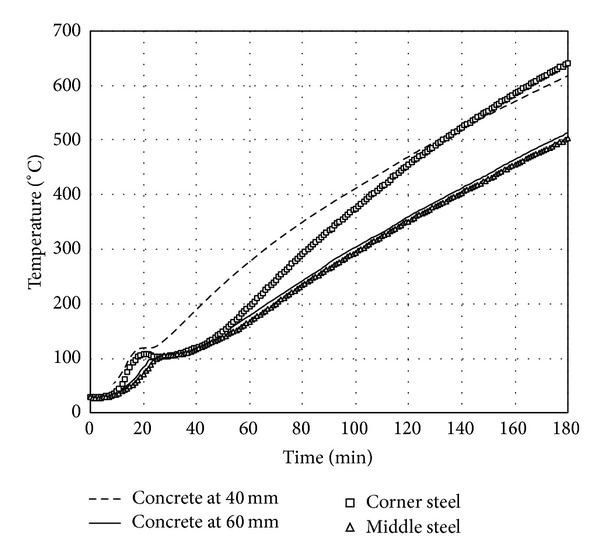
Comparison between temperature evolution in concrete and steel.

**Figure 8:**
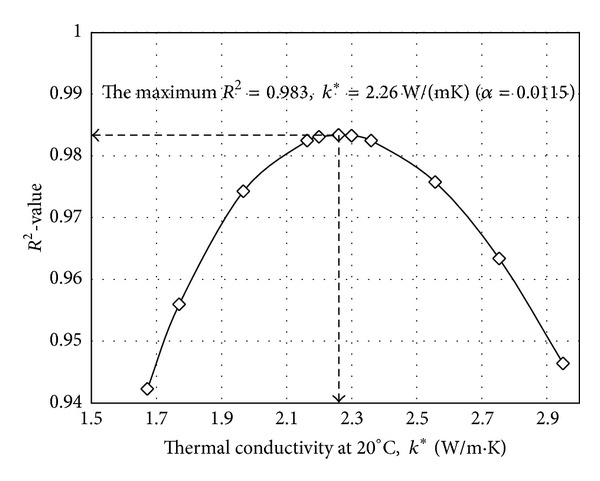
Regression analysis to identify *α* giving the maximum *R* ^2^ value (i.e., minimum error).

**Figure 9:**
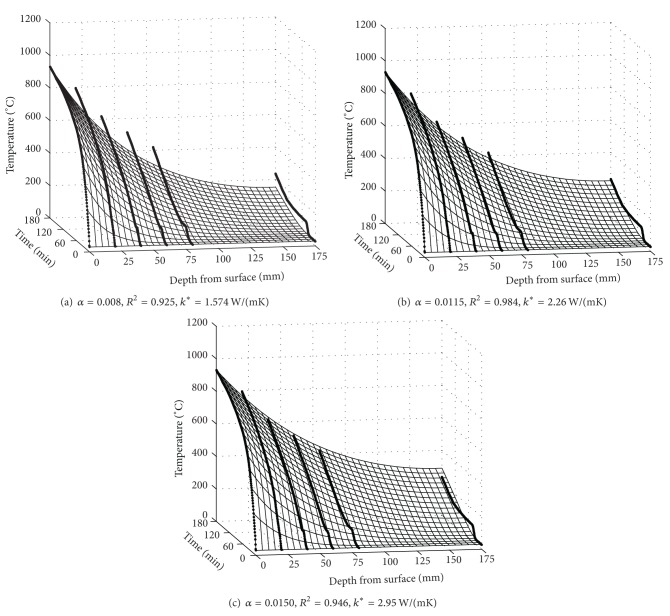
Temperature surfaces according to (a) *α* = 0.008, (b) *α* = 0.0115, the optimal surface with the thermal conductivity *k*_RC_ at 20°C of 2.26 W/(mK) which give the maximum *R* ^2^ value of 0.983, and (c) *α* = 0.0150.

**Figure 10:**
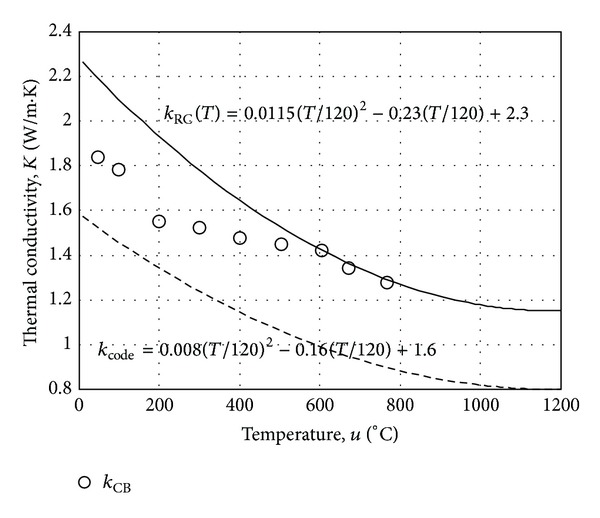
Comparison of thermal conductivity *k*_RC_ (proposed method), *k*_CB_ (concrete brick testing), and *k*_CODE_ (Korean code of practice), respectively.

The time histories of temperatures measured in longitudinal steel bars located at the middle of a side and the corner of the square section are averaged, respectively. The average time histories of temperatures are presented in Figure 6. Because the longitudinal steel bars located at the corner of square section take heat flux from two sides, the temperature of the corner steel bars was always higher than the temperature of steel bars located at the middle of a side. This observation indicates that two-dimensional heat transfer mechanism is necessary to predict steel temperature located at the corner of square section. However, the time history of temperature in the steel located at the middle of a side is almost identical to that in concrete at the depth of 60 mm from the surface as shown in Figure 7. Therefore, the temperatures in steel bars located at the middle of a side of the square section can be estimated to be equal to concrete temperatures at the same depth from surface. Using the time histories of temperatures at the six locations, a regression surface which gives the maximum *R* ^2^ value of 0.983 was achieved with *α* value in (7) of 0.0115 as shown in Figure 8. The corresponding thermal conductivity at 20°C, k* is calculated as 2.26 W/(mK). In Figures 9(a), 9(b), and 9(c), the calculated time histories of temperature variations using the numerical analysis with *α* of 0.008, 0.0115, and 0.015 are compared with the observed time histories of temperature variations, respectively. The time history of temperature variations in Figure 9(b) is obviously the optimal temperature variation. A monotonic sequence can be observed in Figure 9. The increase of thermal conductivity of concrete enables conducting more heat to the center of the square section of RC column. The thermal conductivity of concrete for RC column with respect to temperature *k*_RC_(*T*) is, then, determined using (7) as shown in Figure 10. The thermal conductivity of concrete bricks *k*_CB_ measured at the test temperatures 50°C, 100°C, 200°C, 300°C, 400°C, 500°C, 600°C, 670°C, and 780°C following ASTM C1113 [24] are also presented in Figure 10. At each test temperature, the thermal conductivity of concrete bricks was measured three times and the results were averaged. The thermal conductivity computation with respect to temperature of normal concrete proposed by the Euro code [6] (*k*_code_) presented in (7) with *α* of 0.008 is plotted in Figure 10. As shown in Figure 10, the standard thermal conductivity *k*_CB_ is less than *k*_RC_ until the temperature reaches 600°C. It can also be observed that the difference between the two measurements gets reduced as the temperature increases from 200°C to 600°C. This might be attributed to the fact that *k*_CB_ is the thermal conductivity for concrete with empty pores because water in the capillary pores evaporates during the soak period to enforce the thermal steady-state at test temperature. However, *k*_RC_ is the thermal conductivity for concrete with partially saturated capillary pores, which might be more preferable for practical use. The observations of *k*_CB_ over the temperature of 600°C agree well with *k*_RC_. Moreover, the thermal conductivity *k*_code_ proposed by the design code is less than *k*_RC_. Therefore, it might be necessary to consider the effect of partially saturated capillary pores on the thermal conductivity of concrete for a conservative estimate of RC members' thermal behavior in fire.

### 4.2. Radiant Heat Transfer Ratio of Concrete

**Figure 11:**
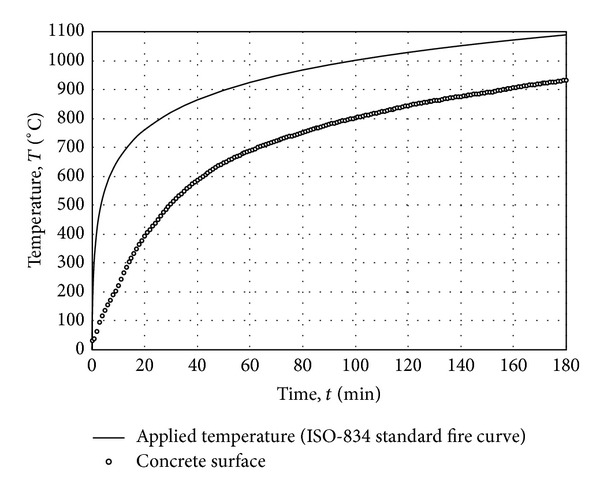
The difference in ISO-834 fire curve and the observed temperature on the surface of RC column confirming the unsteady-state conditions of the test measurements.

**Figure 12:**
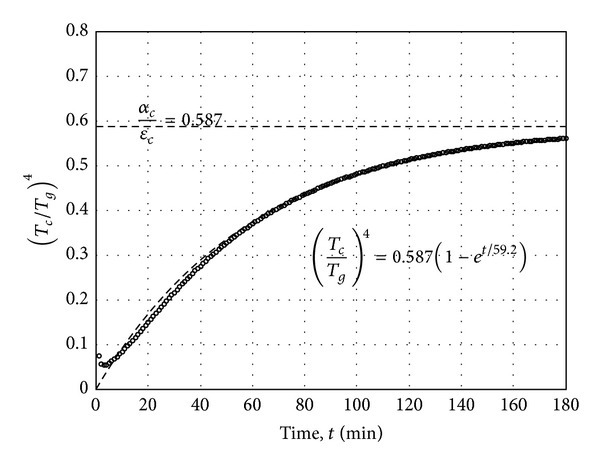
Regression analysis of (*T*_*c*_/*T*_*g*_)^4^ formulation versus time.

Examining the temperature difference between the surface of RC column and ISO-834 standard fire curve shown in Figure 11, it can be observed that the RC column is still in thermal unsteady-state. At the surface of RC column, the heat flux is transferred from fire to the column by both convection and radiation. The thermal equilibrium at the surface is formulated as

(10) $$\begin{array}{c} {\dot{q}}_{c}+{\dot{q}}_{r}=\rho \left\{c\left(T\right)+T\left(t,0\right)\frac{dc\left(T\right)}{dT}\right\}\frac{dT\left(t,0\right)}{dt}\left(\frac{V}{S}\right), \end{array}$$

where ${\dot{q}}_{c}$ and ${\dot{q}}_{r}$ are the heat flux components due to convection and radiation, respectively. *V* and *S* are the volume and the exposure area of the RC column, respectively. According to the amount of transferred heat flux and the surface temperature, the time rate of increase of temperature *dT*(*t*, 0)/*dt* will be determined. The amount of heat flux by convective transfer is given by

(11) $$\begin{array}{c} {\dot{q}}_{c}=h_{c}\left(T_{g}-T_{c}\right), \end{array}$$

where *h*_*c*_ is the coefficient of convective heat transfer. *T*_*g*_ and *T*_*c*_ are the gas temperature in fire test furnace and the surface temperature of the RC column in Kelvin. By assuming that the surrounding gas in fire test furnace is a black body and the RC column is a grey body, the amounts of heat flux by radiant transfer are formulated with the thermal properties of concrete as

(12) $$\begin{array}{c} {\dot{q}}_{r}=\sigma \left(\alpha_{c}T_{g}^{4}-\varepsilon_{c}T_{c}^{4}\right), \end{array}$$

where *σ* is Stefan Boltzmann's constant of 5.67 × 10^−8^ W/(m^2 ^K^4^). *α*_*c*_ is the thermal absorptivity of concrete for the gas in fire test furnace and *ε*_*c*_ is the thermal emissivity of concrete toward the gas. As indicated in (12), the radiant heat transfer keeps until *T*_*c*_^4^ is equal to (*α*_*c*_/*ε*_*c*_) *T*_*g*_^4^. Therefore, when (*T*_*c*_/*T*_*g*_)^4^ is equal to *α*_*c*_/*ε*_*c*_, the radiant heat transfer stops. We name the *α*_*c*_/*ε*_*c*_ ratio, here, as the radiant heat transfer ratio. Considering this aspect, (*T*_*c*_/*T*_*g*_)^4^ with time is formulated in Figure 12. As shown in Figure 12, the change of (*T*_*c*_/*T*_*g*_)^4^ with time decreases as it tends to converge. An exponential equation can be used to approximate the convergence value of (*T*_*c*_/*T*_*g*_)^4^ such as

(13) $$\begin{array}{c} {\left(\frac{T_{c}}{T_{g}}\right)}^{4}=\frac{\alpha_{c}}{\varepsilon_{c}}\left(1-e^{t/\tau }\right). \end{array}$$

**Figure 13:**
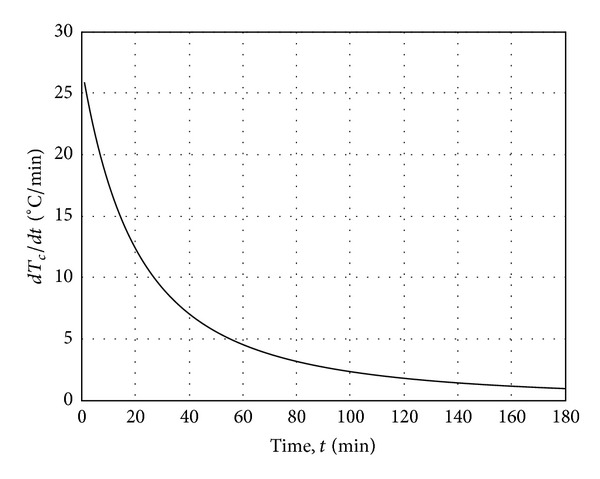
Time rate of temperature increase *dT*(*t*, 0)/*dt* versus time.

Using regression analysis of (13) with the formulation of (*T*_*c*_/*T*_*g*_)^4^ with time, the radiant heat transfer ratio *α*_*c*_/*ε*_*c*_ of 0.587 and *τ* of 59.2 minutes is determined as shown in Figure 12. Considering that the radiant heat transfer ratio *α*_*c*_/*ε*_*c*_ represents the ratio of radiant heat flux inflow to outflow of the RC column, the radiant heat transfer ratio can be used as a reference value to assess fire resistance of RC elements. Concrete with a low the radiant heat transfer ratio *α*_*c*_/*ε*_*c*_ will be capable of preventing thermal radiant heat transfer to the entire structure when exposed to fire. It is interesting to note that although the radiant heat transfer ratio cannot be directly compared with that for solar radiation because of the difference in wave lengths of thermal energy in fire test furnace from that of solar energy, the ratio determined in this study (0.587) is close to the value of 0.568 reported for concrete and based on solar absorptivity of concrete 0.5 over the emissivity of concrete as 0.88 [31]. There have been extensive studies in the literature about the ratio of the solar absorptivity over emissivity for selecting appropriate coating materials for spacecraft [28]. The regression analysis to find the radiant heat transfer ratio *α*_*c*_/*ε*_*c*_ will be effective when the time rate of temperature increase *dT*(*t*, 0)/*dt* in (10) significantly decreases such as in Figure 13. It is notable that the radiant heat transfer ratio *α*_*c*_/*ε*_*c*_ is developed due to the black body assumption of the gas surrounding the RC column in fire test furnace. Moreover, the thermal radiation of compartment walls making up the fire test furnace is also neglected [32]. Therefore, if the information about the combined emissivity *ϕ* of gas and compartment walls in test fire furnace is available [32], the radiant heat transfer ratio *α*_*c*_/*ε*_*c*_ can be divided by *ϕ* and can be used as a criterion for concrete for fire safety assessment.

## 5. Conclusions

An inverse methodology to find the thermal conductivity from time history of temperature variations in the thermal unsteady-state is proposed. A square RC column with cross section of 350 mm by 350 mm and the height of 1500 mm was tested in order to evaluate the time history of temperature variations following ISO-834 standard fire curve. Using a numerical solution for thermal equilibrium model in the unsteady-state condition, the thermal conductivity of concrete for the RC column was determined in quadratic form as *k*(*T*) = 0.0115{(*T*/120)^2^ − 20(*T*/120) + 200} in W/(mK) for the temperature range from 20°C to 1200°C. Thermal conductivity of concrete was conservatively estimated using the proposed method when compared with the thermal conductivity of concrete bricks measured using ASTM standards. Moreover, another aspect of fire safety assessment was evaluated from the difference of the applied temperature (ISO-834 standard fire curve) and the surface temperature in fire test furnace. The radiant heat transfer ratio representing concrete absorptivity over emissivity *α*_*c*_/*ε*_*c*_ ratio was determined from regression analysis of (*T*_*c*_/*T*_*g*_)^4^ formulation with time. It can be observed that concrete with relatively low radiant heat transfer ratio can protect structures during fire by preventing thermal radiant heat transfer to the structures from fire. It is suggested that the radiant heat transfer ratio shall be used as a concrete criterion for fire safety assessment.

## Figures and Tables

**Table 1:** Concrete mixture (kg/m^3^).

W/C	Water	Cement	Sand	Gravel
0.55	175	320	827	939

## Nomenclatures

*c*(*T*): Specific heat at the temperature of *T*
*h*_*c*_: The coefficient of convective heat transfer
*i*: The number of time steps
*j*: The number of distance steps
*k*(*T*): Thermal conductivity at the temperature of *T*
${\dot{q}}_{c}$: Heat flux components due to convection
${\dot{q}}_{r}$: Heat flux components due to radiation
*S*: The exposure area of RC column
*c*(*T*): Specific heat at the temperature of *T*
*k*(*T*): Thermal conductivity at the temperature of *T*
*T*_0_: Ambient temperature
*T*_*c*_: The surface temperature of RC column
*T*_*g*_: Gas temperature in fire test furnace
*T*(*t*, *x*): Temperature of concrete at the distance *x* from the surface at time *t*
*V*: The volume of RC column
*α*: The variable for the thermal conductivity in(7)
*α*_*c*_: Thermal absorptivity of concrete for the gas in fire test furnace
*β*: The coefficient for the specific heat in (8), which is fixed at 4.0
*ε*_*c*_: Thermal emissivity of concrete toward the gas
Δ*t*: The step sizes for time
Δ*x*: The step sizes for distance
*ρ*: Density of concrete
*σ*: Stefan Boltzmann constant, which is 5.67 × 10^−8^ W/(m^2^K^4^).
